# Numerous insertions of mitochondrial DNA in the genome of the northern mole vole, *Ellobius talpinus*

**DOI:** 10.1007/s11033-023-08913-4

**Published:** 2023-12-29

**Authors:** Kristina Kuprina, Antonina Smorkatcheva, Anna Rudyk, Svetlana Galkina

**Affiliations:** 1 Institute of Botany and Landscape Ecology, University of Greifswald, Soldmannstr. 15, Greifswald, 17489 Germany; 2 Department of Vertebrate Zoology, Saint Petersburg State University, Universitetskaya nab. 7/9, Saint Petersburg, 199034 Russia; 3 Department of Genetics and Biotechnology, Saint Petersburg State University, Universitetskaya nab. 7/9, Saint Petersburg, 199034 Russia

**Keywords:** NUMTs, Mitochondrial pseudogenes, Heteroplasmy, Arvicolinae, Rodentia

## Abstract

**Background:**

*Ellobius talpinus* is a subterranean rodent representing an attractive model in population ecology studies due to its highly special lifestyle and sociality. In such studies, mitochondrial DNA (mtDNA) is widely used. However, if nuclear copies of mtDNA, aka NUMTs, are present, they may co-amplify with the target mtDNA fragment, generating misleading results. The aim of this study was to determine whether NUMTs are present in *E. talpinus*.

**Methods and results:**

PCR amplification of the putative mtDNA *CytB*-D-loop fragment using ‘universal’ primers from 56 *E. talpinus* samples produced multiple double peaks in 90% of the sequencing chromatograms. To reveal NUMTs, molecular cloning and sequencing of PCR products of three specimens was conducted, followed by phylogenetic analysis. The pseudogene nature of three out of the seven detected haplotypes was confirmed by their basal positions in relation to other *Ellobius* haplotypes in the phylogenetic tree. Additionally, ‘haplotype B’ was basal in relation to other *E. talpinus* haplotypes and found present in very distant sampling sites. BLASTN search revealed 195 NUMTs in the *E. talpinus* nuclear genome, including fragments of all four PCR amplified pseudogenes. Although the majority of the NUMTs studied were short, the entire mtDNA had copies in the nuclear genome. The most numerous NUMTs were found for *rrnL*, *COXI*, and D-loop.

**Conclusions:**

Numerous NUMTs are present in *E. talpinus* and can be difficult to discriminate against mtDNA sequences. Thus, in future population or phylogenetic studies in *E. talpinus*, the possibility of cryptic NUMTs amplification should always be taken into account.

**Supplementary Information:**

The online version contains supplementary material available at 10.1007/s11033-023-08913-4.

## Introduction

The presence of sequences with significant homology to mitochondrial DNA (mtDNA) has been established in the nuclear genome of many eukaryotes [1, 13, 16, 25, 33, 34, 50]. These sequences (*Nu*clear *m*i*t*ochondrial sequences or NUMTs, also called mtDNA pseudogenes) can be of various amounts and lengths, and most of them are non-functional degenerate copies of various mtDNA fragments [6]. Being numerous and predominantly selectively neutral, these “relics of ancient DNA” are valuable molecular markers in evolutionary biology that can be used in rooting and resolving phylogenetic reconstructions and estimating nuclear mutation rates [5].

Information on the distribution of NUMTs integration sites has been accumulating (e.g., [18, 21]). In organisms with well-developed genome assemblies, NUMTs can be identified bioinformatically through targeted searching for mtDNA inserts in nuclear genome sequences. At the early stage of genome sequencing and assembly studies, NUMTs are detected occasionally as artefacts, when nuclear DNA is co-amplified in PCR using mtDNA-specific primers [7, 8, 11, 22, 32]. Therefore, from a practical point of view, NUMTs are a potential source of contamination during PCR amplification of mtDNA fragments. The risk of pseudogene co-amplification further increases with ‘universal’ primers. NUMTs are known to be a source of errors in phylogeny and phylogeography reconstruction [2, 20, 27, 30, 39, 41, 45] DNA barcoding [8, 44], and disease association with mutations in mtDNA [46, 48]. Analyse of mtDNA sequence diversity is a popular tool for assessing population structure and dynamics, while differentiation of an authentic mitochondrial sequence from its pseudogene is critical for the correct interpretation of haplotype diversity. Therefore, identification of NUMTs in the genomes of the species used as models in population studies is of great importance.

Mole voles (*Ellobius*, Arvicolinae, Cricetidae) are highly specialised subterranean rodents inhabiting the grasslands of Eurasia. These rodents represent an attractive model in population ecology studies due to their lifestyle, sociality and unusual life-history traits, e.g., slow growth and development, delayed sexual maturity, and longevity extreme among voles [19, 22, 31, 37]. However, until nowadays, only *сytochrome B* (*CytB*) sequences were investigated in several population genetic studies of this genus [12, 15, 43]. Meanwhile, the D-loop region has several advantages as a molecular marker for assessment of genetic diversity and structure: it is non-coding (therefore, we can presume they are selectively neutral) and the most variable part of mtDNA, mainly due to mutations rather than recombination [17, 40, 42]. During the development of a primer combination to amplify a fragment of D-loop region for the northern mole vole (*Ellobius talpinus*), we detected multiple occurrences of double peaks in the chromatograms. We inspected cloned PCR products via sequencing and phylogenetic analysis to test the hypothesis that the nuclear genome of the northern mole vole does contain NUMTs homologous to D-loop region and *CytB* fragments. Additionally, the genome assembly of *E. talpinus* was analysed to identify other NUMTs for use in evolutionary studies and checking their potential interference against the existing studies of mitochondrial genetic variation.

## Materials and methods

**Fig. 1:**
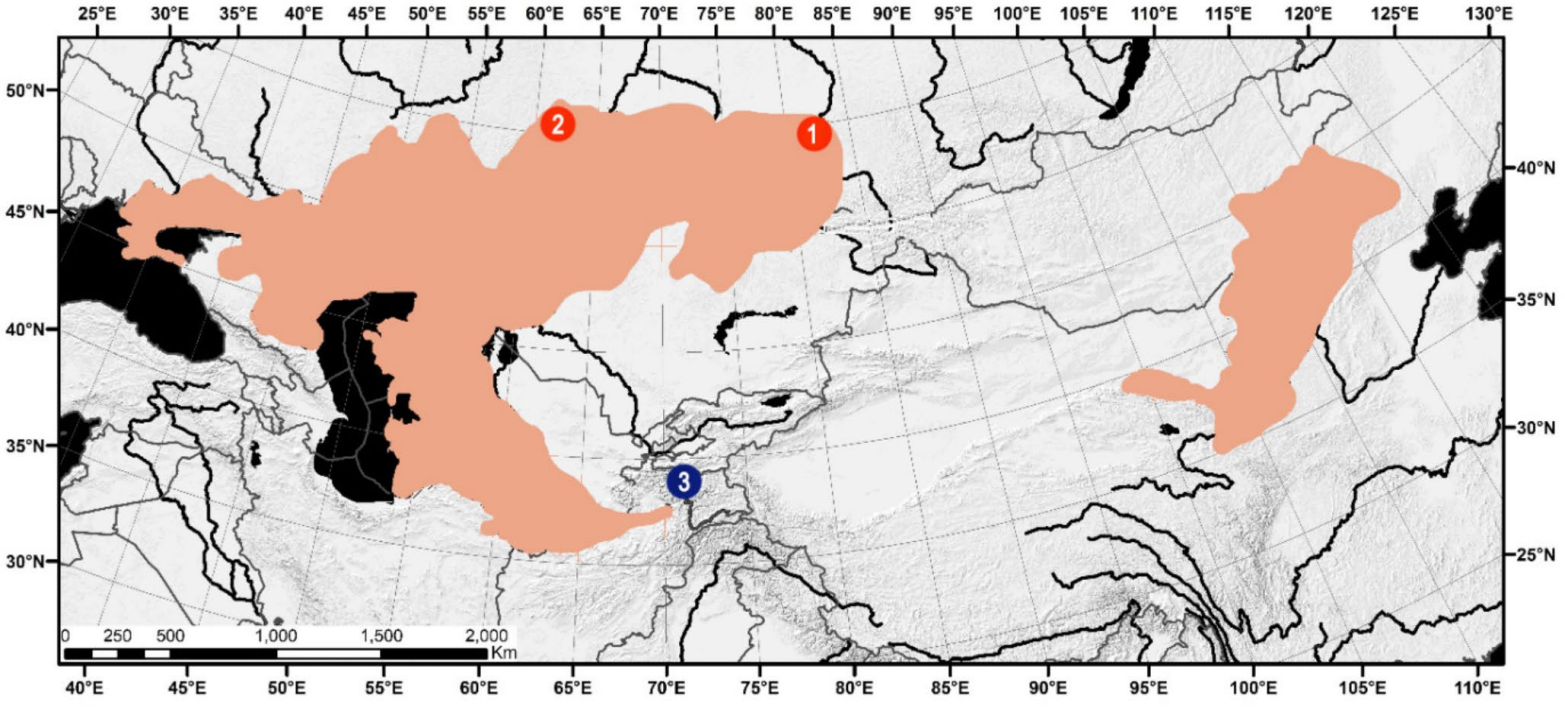
Species distribution map of *Ellobius talpinus* with sampling sites: 1 - Novosibirsk population (n = 56); 2 - Chelyabinsk population (n = 1), and 3 - sampling site of *E. tancrei* (n = 3). Map (modified) from [23]

The northern mole voles (*Ellobius talpinus*) were live-trapped in the Novosibirsk (n = 56) and Chelyabinsk regions (n = 1) of Russia as part of population studies (Fig. 1). Three Zaisan mole voles (*E. tancrei)* from southwestern Tajikistan were also included in this study. The animals were marked by toe clipping, and the phalanges were further stored in 96% ethanol for genetic analysis. All applicable institutional guidelines for the care and use of animals were followed. After the toe clipping, animals were released into a burrow where they were captured. The study was conducted under the ethical clearance from the Ethical Committee of Saint Petersburg State University (Statement no. 131-03-9 issued on 22 November, 2021) in accordance with the National Research Council (2011).

DNA was extracted according to the standard phenol-chloroform procedure [36]. For PCR, a pair of ‘universal’ primers ArvF 5’-GCCTCAATCGCCTACTTCAC-3’ and ArvR 5’-GGCTGATTAGACCCGTACCA-3’ was designed by aligning the published mtDNA sequences of Arvicolinae (NCBI: FJ502319, JX996084, JX996089, AF192739, AF348371 - AF348384, AF267284, HQ395088, DQ198847, GQ452102). The desired fragment was expected to be 670 bp long and include 84 bp of *CytB* gene, two tRNA genes, and a part of D-loop which comprised the first hypervariable region (Supplementary Fig.1). PCR with these primers was conducted for all *Ellobius* specimens. PCR reaction mixture (20 µl) contained 1X Taq-buffer, 1 U HotStart Taq polymerase, 1.5 mM MgCl_2_ (all from Axon Labortechnik, Germany), 4 µM dNTPs, 0.2 µM each primer, and 60 ng of DNA as a template. Amplification was done in a thermocycler MJ Mini (Bio-Rad Laboratories, USA) according to the following program: (i) 13 min at 95 °C; (ii) 35 cycles of amplification consisting of 45 s at 95 °C, 30 s at 55 °C and 30 s at 72 °C; (iii) 3 min final extension at 72 °C. All PCR products including no template controls were analysed by agarose gel electrophoresis. For three *E. talpinus* from the Novosibirsk region, PCR products were purified on silica magnetic beads (Sileks, Russia) and cloned using the ”InsTAclone PCR Cloning Kit“ (Thermo Fisher Scientific, USA). PCR amplicons and cloned insert fragments were sequenced on an ABI Prism 3500xl analyser (Thermo Fisher Scientific, USA). All laboratory procedures were conducted using filter tips; no-template controls were included in every PCR and sequenced. To additionally verify the absence of contamination, 8 samples of *E. talpinus* were extracted and amplified with new reagents in a different laboratory.

Sanger sequencing chromatograms were visually inspected using Chromas 2.6.6 (Technelysium Pty Ltd, Australia), and in the case of double peaks, the highest peak was used to determine the nucleotide. Forward and reverse reads were compared pairwise to exclude the possible sequencing errors. All obtained sequences were manually aligned using MEGA X [24], and the resulting haplotypes were checked for specificity using BLASTN. MEGA X software was also used for checking the reading frame in a fragment of *CytB*.

To check whether NUMTs had been co-amplified, a phylogenetic analysis of all obtained haplotypes was conducted. When phylogenetic relationships within a taxon are well known, pseudogenes can often be detected by the atypical length of their branches and irregular topology [3, 4, 9, 38], since the mutation rate in NUMTs is about ten times lower compared to mtDNA [5, 26]. D-loop sequences of *E. tancrei*, steppe lemming, *Lagurus lagurus*, yellow steppe lemmings, *Eolagurus luteus*, and European water vole, *Arvicola amphibius*, were included in the analysis; the large-eared vole, *Alticola macrotis* and the bank vole, *Myodes glareolus* were used to root the tree. The sequences for *E. tancrei* were obtained in this work using ‘universal’ primers, the rest of the sequences were retrieved from GenBank (Supplementary Table 1). We used 47 bp fragment of *cytochrome B*, *tRNA-Thr*, *tRNA-Pro*, and 450 bp D-loop fragment, in total 631 bp corresponding to positions 15,212–15,842 bp of the *E. talpinus* mtDNA (NCBI: NC_054160). The best-fit substitution model (HKY + G) was found using MEGA X. Accordingly, the combination of nst = 6, a discrete Gamma distribution (+ G) with 4 rate categories with assuming that a certain fraction of sites is evolutionarily invariable were used for Bayesian analysis. The phylogenetic tree was built using MrBayes v.3.2 [35] (10,000,000 generations, 25% burn-in) and visualised in FigTree v.1.4.3 (tree.bio.ed.ac.uk/software/figtree/).

The presence of pseudogenes in the nuclear genome of *E. talpinus* was verified by mapping sequencing reads from the SRR3497471 NCBI SRA database to the sequence of each of six haplotypes. At first we assembled a mitogenome for sample SAMN04317029 (www.ncbi.nlm.nih.gov/biosample/SAMN04317029), and we excluded all mitochondrial reads from the subsequent analysis. Mapping the remaining reads to haplotypes was performed using the ‘Map to reference’ option in Geneious Prime (www.geneious.com/) with strong selectivity (maximum 1% of gaps and 1% of mismatches per read were allowed). We selected split reads where a part of the read is mapped to the haplotype and the other part half to the nuclear genome. Such split reads were used in BLAST search to identify the specific contigs in the NCBI whole-genome sequencing (WGS) database GCA_001685095.1 corresponding to the reference genome ETalpinus_0.1 (www.ncbi.nlm.nih.gov/datasets/taxonomy/329620/) and to define the breakpoints. The presence of other NUMTs in the *E. talpinus* nuclear genome was evaluated through BLASTN search on the only available genome assembly GCA_001685095.1. We split *E. talpinu*s mtDNA reference sequence (NCBI: NC_054160) into 16 non-overlapping segments of 1000 bp plus one fragment of 637 bp, and aligned them against GCA_001685095.1. All contigs found were additionally checked for the presence of NUMTs through alignment to NC_054160 using the ‘Align whole genomes’ option (Mauve chromosome alignment algorithm) implemented in Geneious Prime software. Annotation of contigs, including NUMTs lengths, their start and end positions, and the corresponding sites in the *E. talpinus* mtDNA NC_054160 was also done in Geneious Prime (Supplementary Table 2).

## Results

Using ‘universal’ primers ArvF and ArvR, we amplified the PCR products from 56 Novosibirsk samples and one Chelyabinsk sample of *E. talpinus*. All amplicons produced single bands in electrophoresis. They were sequenced, and four haplotypes (A, B, C, and D) were detected. The haplotypes A, C and D had 1 (A vs. C), 2 (A vs. D) or 3 (C vs. D) variable sites; however, haplotype B differed from A, C and D in 33–35 positions. Most of the Novosibirsk samples (64%) as well as the sample from the very distant Chelyabinsk region had haplotype B. For a number of samples, the sequencing resulted in chromatograms containing double peaks at more than 30 nucleotide sites. Clean laboratory practice combined with absence of PCR products in the agarose gel (Supplementary Fig. 2a) and in the chromatograms for no-template controls confirms the absence of contamination; however, double peaks of different heights (Supplementary Fig. 2b) in > 90% of the working chromatograms persisted, indicating potential NUMT co-amplification.

Following the demonstration of abnormally diverged sequences generated by ‘universal’ primers, we cloned the PCR products of three Novosibirsk samples and sequenced several (3, 5, and 16) plasmids per individual. No double peaks were found in the chromatograms. We identified haplotypes A, B, C and D, and three additional ones: E, F, and G. Haplotype B was found in all three samples along with either A, or C, or D. Haplotypes E, F and G differed from haplotype A in 89, 62 and 66 bp, respectively. The BLAST search of the haplotypes found the closest similarity with the mtDNA of *E. talpinus*. In the *CytB* fragment, neither an additional stop codon nor any frame shifts were detected for any of the haplotypes. All haplotypes obtained by ‘universal’ primers were uploaded to GenBank (NCBI: OR662053 (*E. tancrei*), OR662050 - OR662052 (*E. talpinus* haplotypes A, C, and D), OR662054 - OR662057 (*E. talpinus* pseudogenes B, E, F, and G)).

**Fig. 2:**
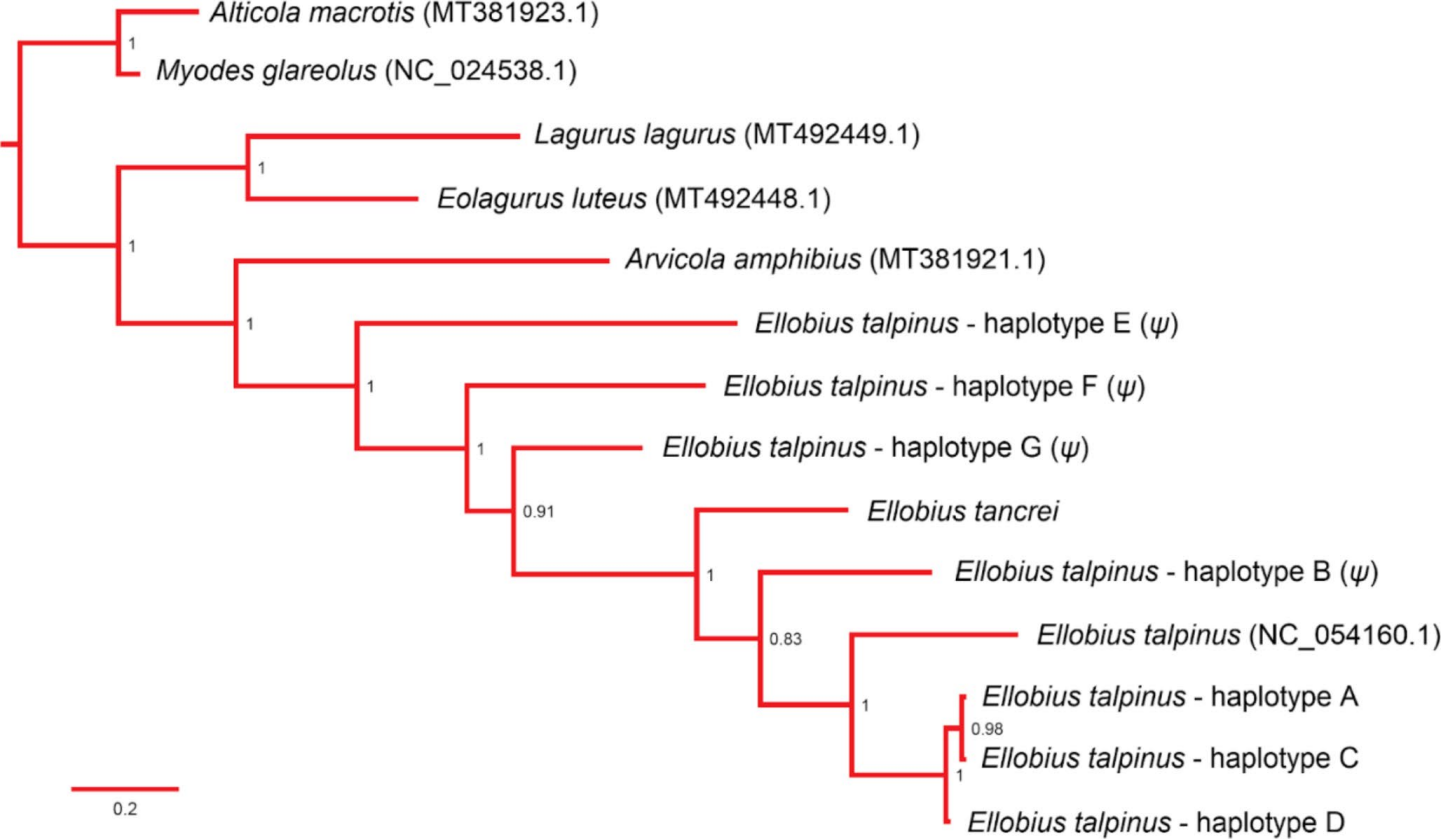
Bayesian tree of 7 Arvicolinae species based on the 631 bp fragment of mtDNA, including the haplotypes of *E. talpinus* and *E. tancrei*, amplified with ‘universal’ primers. Putative pseudogenes are indicated by *ψ*. The sequences not obtained in this study are shown with the NCBI accession numbers in brackets

Phylogenetic analysis of the detected *Ellobius* haplotypes together with corresponding sequences of the selected arvicolines revealed the basal position of *E. talpinus* haplotypes E, F, and G relative to the other haplotypes of the genus Ellobius (Fig. 2). The remaining *E. talpinus* haplotypes together with *E. tancrei* formed a monophyletic group.

To check whether a primer with greater specificity can decrease or eliminate co-amplification of revealed pseudogene(s), a new primer, EtalpF (5’-TCAAGAAGGAAGGACCTACCC-3’), was designed based on the results of phylogenetic analysis and mitogenome of *E. talpinus* (NCBI: NC_054160.1). This primer, together with ArvR, was used to amplify mtDNA D-loop of 16 Novosibirsk samples, which previously showed haplotype B. All of the obtained sequences were unambiguous. Haplotype B was found in two samples, while the rest had haplotypes A or D (haplotype C was not detected due to a shorter size of the amplified fragment).

**Fig. 3:**
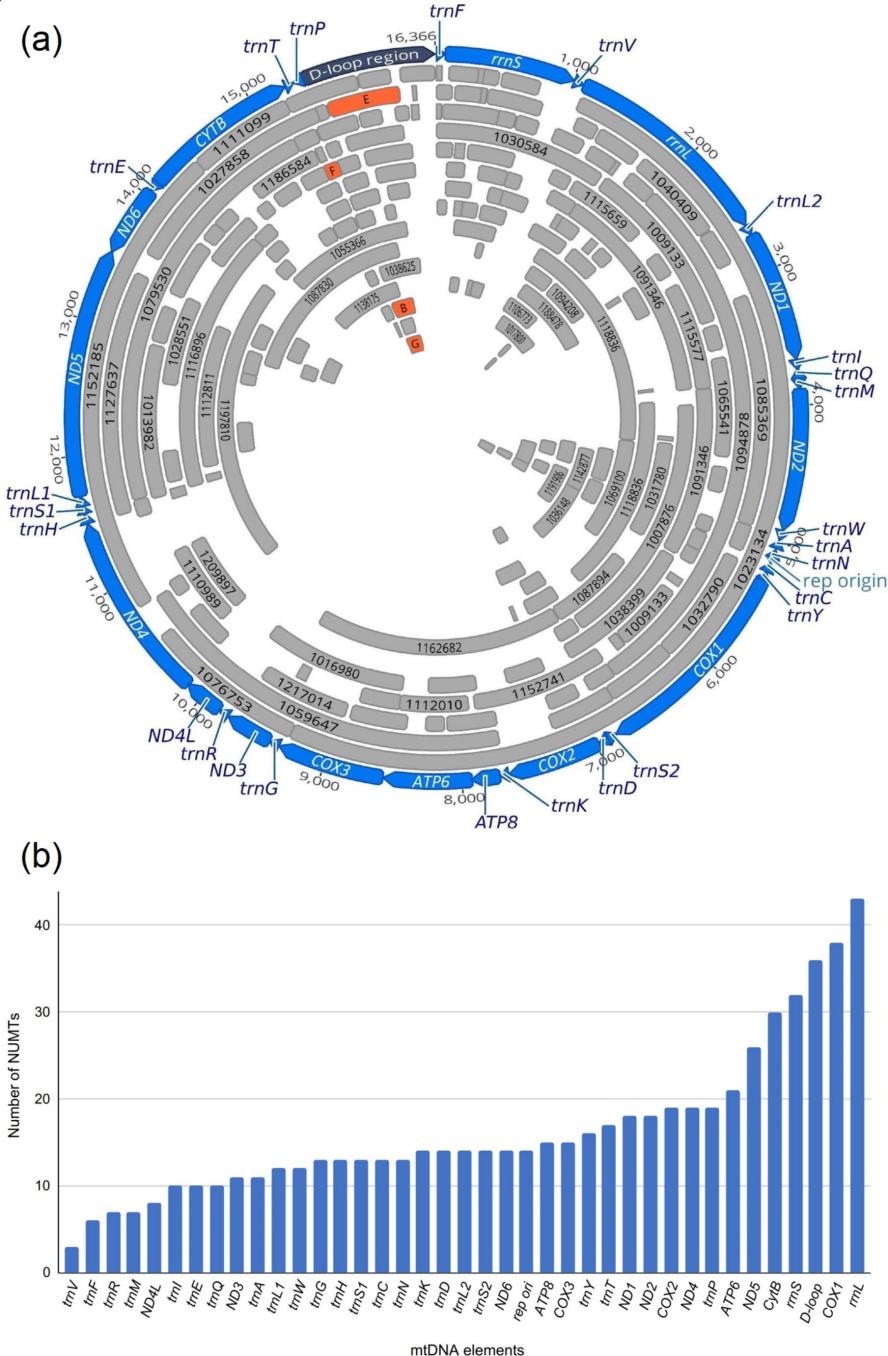
Distribution of NUMTs corresponding to various elements of the *E. talpinus* mitochondrial genome. (a) Circular representation of *E. talpinus* mtDNA (blue circle), which contains the genes for 13 energy pathway proteins, two rRNAs, and 22 tRNAs, and the replication origin and D-loop control region. In mtDNA circle, the grey lines indicate NUMT insertions found in WGS contigs from the GCA_001685095.1 genome database. Letters E, F, B and G label the contigs containing fragments of pseudogenes E, F, B, G, correspondingly. The length of each line reflects the length of the corresponding NUMT. The longest NUMTs are marked with the numbers of the corresponding contigs. (b) The number of contigs bearing NUMTs for each of the 39 mtDNA elements including replication origin element and D-loop region. Each contig was counted even if it contained a fragment of an element

To test bioinformatically whether the sequences corresponding to putative pseudogenes are present in the nuclear genome, we mapped the sequencing reads from the *E. talpinus* SRR3497471 NCBI SRA database to each of the six revealed haplotypes. After exclusion of all mitochondrial reads, no additional reads aligned to haplotypes A, C, and D. As for haplotypes B, E, F, and G, we identified split reads, which allowed the detection of several contigs in the *E. talpinus* GCA_001685095.1 genome database. When searching for 1 kb fragments of mtDNA sequences in the GCA_001685095.1, we found 240 WGS contigs (Supplementary Table 2); 45 were found to be entirely mitochondrial; they were annotated but excluded from the subsequent counting (Supplementary Table 2). In the remaining 195 contigs, the boundaries of a mtDNA fragment copied in a NUMT were determined by the alignment between the corresponding contig and the NC_054160.1 mitogenome (Fig. 3a, Supplementary Table 2). NUMTs ranged in size from 31 to 4536 bp, median being 315 bp (Supplementary Fig.4). The majority of NUMTs were short insertions: 65.6% were less than 500 bp in size. NUMTs for *ND5*, *CYTB*, *COXI* protein-coding genes as well as for D-loop and both rRNAs were the most copious. In contrast, NUMTs for *ND4L* and *ND3* genes were rare (Fig. 3b).

## Discussion

In this study, we present two arguments supporting the hypothesis that the nuclear genome of *E. talpinus* contains numerous insertions of various mtDNA fragments. The first argument comes from a practical problem related to amplification of D-loop sequences using ‘universal’ primers for the northern mole vole. The frequent detection of double peaks in the *CytB*-D-loop fragment chromatograms and the presence of ‘haplotype B’ in animals from very distant sampling sites (located more than 1300 km from each other, Fig. 1) gave rise to the NUMTs co-amplification hypothesis. Molecular cloning of PCR-amplicons supported this hypothesis indicating the presence of haplotype B, together with one of three other previously revealed haplotypes (A, C, or D) in each individual. Moreover, cloning discovered three extra haplotypes (E, F, and G) whose pseudogene nature was confirmed by their basal positions in relation to the other obtained *Ellobius* haplotypes in the phylogenetic tree (Fig. 2) and by finding sequencing reads comprising both mitochondrial and non-mitochondrial DNA fragments (Supplementary Fig.5). The second evidence comes from the results of our BLAST search in *E. talpinus* GCA_001685095.1 genome database showing that 195 WGS contigs contain sequences of mitochondrial origin of various lengths. Thus, the genome of the northern mole vole is not an exception among other voles in terms of the NUMT content [1, 3]. This is the first report on NUMTs in the nuclear genome of *Ellobius*.

Some cases of predominant amplification of pseudogenes by universal primers were described in the past [11, 49]. In our case, the conservative primer ArvF designed using the sequences of other Arvicolinae species had two non-specific to *E. talpinus* sites. These sequences lead to amplification of haplotype B with high probability, and, in a minority, haplotypes E, F, and G. In this work, in order to minimise the presence of pseudogenes in the PCR products, we designed a new primer, EtalpF, which considerably diminished, although did not completely eliminate, the amplification of the pseudogene. In a recently initiated population genetic study of mole voles from the Saratov Region, we obtained 75 sequences using the EtalpF primer (Rudyk et al., in preparation). Several chromatograms showed double peaks at certain nucleotide sites; their positions indicated co-amplification of pseudogene B together with the authentic D-loop region fragment. Moreover, one of the unambiguous sequences completely corresponded to pseudogene B. To discriminate between pseudogenes and orthologous coding mitochondrial sequences, the reading frame and stop codons can be inspected. However, this method is unsuitable for detecting pseudogenes of the D-loop region because of the non-coding nature of the fragment. So, we strongly urge that when analysing mitochondrial polymorphism in these rodents, the possibility of cryptic NUMTs amplification should always be kept in mind.

When double peaks on chromatograms and/or unusually common haplotype(s) are detected for D-loop region sequences, checking the phylogenetic relationships of all identified haplotypes in order to detect and exclude haplotypes with an anomalous basal position has been recommended [2, 4, 7, 9]. Our phylogenetic reconstruction of seven haplotypes of *E. talpinus* (obtained with the “universal” primers) along with the corresponding fragments for six other arvicoline species showed that three haplotypes of *E. talpinus* (E, F, and G) had a basal position relative to the divergence of two *Ellobius* species, indicating the early origin and nuclear source of sequenced fragments (Fig. 2). The position of haplotype B in the *Ellobius* clade did not receive high support, but was basal relative to the clade encompassing haplotypes A, C, D, and *E. talpinus* NC_054160.1. This basal position of haplotype B together with its wide geographical distribution and most frequent occurrence indicate the ubiquitous and pseudogenic nature of haplotype B. The aligning of haplotypes B, E, F, and G with the reads from the *E. talpinus* nuclear genome supported our characterization of them as NUMTs.

In our work, we identified 195 NUMTs by aligning the *E. talpinus* mitochondrial genome on the nuclear genome using BLASTN (Supplementary Table 2). The majority of NUMTs were short insertions consistent with ongoing selection against large NUMTs [10]. According to our estimation, the absolute and relative to the nuclear genome amount of NUMTs in the northern mole vole is 125.03 kb or 0.004%. This is two times more than found in another rodent, *Mus musculus* (Murinae, Muridae) (37.67 kb, 0.002%) [6]. This discrepancy can be explained by the insufficient completeness of the available *E. talpinus* genome used in the study (number of contigs 350,460, N50 = 15.2 kb, www.ncbi.nlm.nih.gov/datasets/genome/GCA_001685095.1/). At the same time, among the rodent genomes, the median number of NUMTs ranges from 168 to 11,930 (median 477), being in the Arvicolinae voles 625 (*Microtus agrestis*), 334 (*M. ochrogaste*r), 2,952 (*M. arvalis*), 290 (*Arvicola amphibius*), and 502 (*Myodes glareolus*) [1]. Thus, our modest 195 NUMTs found in *E. talpinus* may be considered a reliable number for these small rodents. Further improvement of the northern mole vole genome assembly will undoubtedly clarify the situation.

We demonstrated that in *E. talpinus*, the entire mtDNA is involved in NUMTs. The most numerous NUMTs were found for *ND5*, *CYTB*, *COXI*, *rrnL*, *rrnS*, and D-loop region; *ND4L*, *ND3* and genes for some tRNAs seldom produce NUMTs (Fig. 3a). Interestingly, *CYTB*, D-loop region, *rrnS* and *rrnL* elements are adjacent, while ND4L and ND3 are situated at the opposite pole (Fig. 3a). None of the previous studies appear to have found local transfer preferences across the mitochondrial genome in rodents [1, 2]. However, in humans, mtDNA breakpoints were found to be more common to the non-coding D-loop region compared to other sites, and were less likely to involve ND3 and ND4L genes [10].

The issue of the D-loop region contribution to NUMTs is still under discussion. Mourier et al. [29] observed a deficiency in the D-loop region derived NUMTs (in humans) but attributed that to the difficulty of NUMT detection in this rapidly evolving region. At the same time, mtDNA D-loop fragments were found in the genomes of rodent species [3]. The presence of D-loop derived sequences in the nuclear genome is indicative of DNA-mediated rather than RNA-mediated NUMT insertions, since the control region has no intermediate RNAs [14, 47]. The current plausible explanation of the emergence of mitochondrial pseudogenes involves mtDNA transcription and associated replication occurring in the D-loop region. This is in line with the recent description of mitochondrial apoptosis mechanism mediated by the BCL-2 family proteins BAK and BAX inducing outer mitochondrial membrane permeabilization through large pores formation [28]. These BAK/BAX macropores allowed the inner mitochondrial membrane to penetrate into the cytosol, taking with it the components of the mitochondrial matrix, including the mitochondrial genome. Once in the cytoplasm, mitochondrial DNA can, under certain circumstances, integrate into the nuclear genome. Although it is unlikely that NUMTs are functional per se due to differences between the nuclear and mitochondrial genetic codes [5], it can be assumed that in rare cases they may perform a functional role as regulatory elements or through the creation of new exons.

## Conclusion

Multiple NUMTs that are difficult to discriminate against mtDNA sequences were detected in the genome of *E. talpinus*. A combination of primers for preferential amplification of mitochondrial fragment of the *CytB*-D loop region has been developed for the future population genetic studies of this species. For any mitochondrial genetic marker, it is strongly recommended to mind the possibility of cryptic NUMTs amplification in *E. talpinus*.

## Electronic supplementary material

Below is the link to the electronic supplementary material.

Supplementary Material 1

## Data Availability

The data generated and/or analysed during the current study are available from the corresponding author upon request.
