# Supplementary material for: Numerous insertions of mitochondrial DNA in the genome of the northern mole vole, Ellobius talpinus
Source: Mol Biol Rep. 2023 Dec 29;51(1):36. doi: 10.1007/s11033-023-08913-4 (PMC10756869; doi:10.1007/s11033-023-08913-4)
Supplement: Supplementary file 1 — Supplementary Material 1 [file 11033_2023_8913_MOESM1_ESM.docx]

Supplementary material

**Numerous insertions of mitochondrial DNA in the genome**

**of the northern mole vole, *Ellobius talpinus***

Kristina Kuprina^1^, Antonina Smorkatcheva^1^, Anna Rudyk^1^, Svetlana Galkina^2^

Table of content

**Figure S1.** Map of the ArvF, EtalpF, ArvR primers positions used for PCR amplification of a mtDNA fragment in the northern mole vole *E. talpinus*.

**Figure S2.** Example of PCR products detection and analysis by electrophoresis in agarose gel and Sanger sequencing.

**Supplementary Figure S3.** Alignment of haplotypes A, C, D and pseudogenes B, E, F, G to fragment 15213-15834 of the *Ellobius talpinus* reference mt DNA sequence NC_054160

**Supplementary Table S1.** List of Arvicolinae mtDNA D-loop sequences used for the phylogenetic analysis

**Supplementary Table S2.** List of contigs from the *E. talpinus* genome database GCA_001685095.1 containing NUMTs.

**Supplementary Figure S4.** Size distribution of NUMTs in *E. talpinus*.

**Figure S1.** Map of the ArvF, EtalpF, ArvR primers positions used for PCR amplification of a mtDNA fragment in the northern mole vole *E. talpinus*. The primers are shown with yellow blocks above the *E. talpinus* reference mtDNA sequence NC_054610 (Bondareva et al., 2020). ArvR primer is common for 670 bp and 519 bp PCR products.


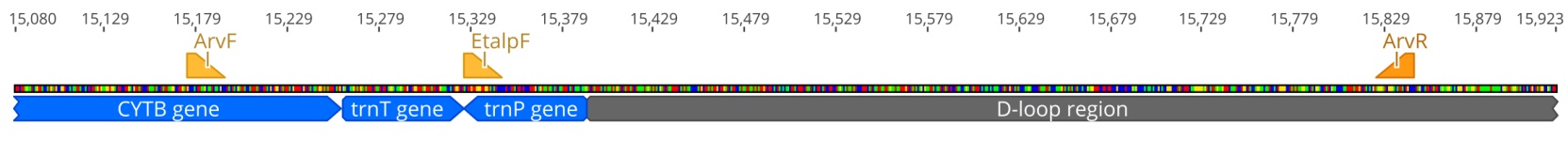


**Figure S2.** Example of PCR products detection and analysis by electrophoresis in agarose gel and Sanger sequencing. (A) Example photo of 1.5% agarose gel after electrophoresis of 6 samples of *E. talpinus* (S1 - S6) and of no template control (NTC). (B) A chromatogram fragment with double peaks, representing the mixture of products and indicative for potential co-amplification of sequences of mitochondrial and nuclear origin.


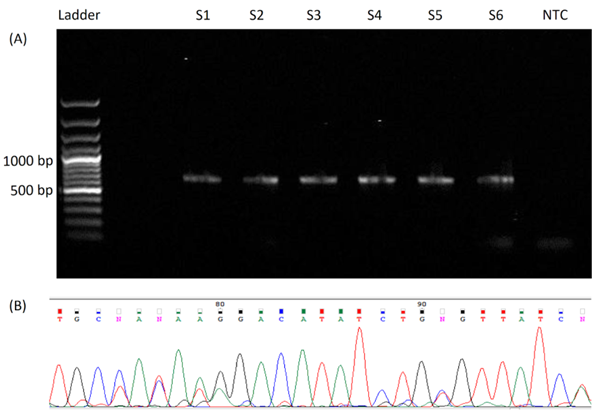


**Supplementary Figure S3.** Alignment of haplotypes A, C, D and pseudogenes B, E, F, G to fragment 15213-15834 of the *Ellobius talpinus* reference mt DNA sequence NC_054160

15222 15232 15242 15252 15261

| | | | |

NC_054160 15213 ACCAATCGCAGGCATAATCGAAAACGACATCTTAGATATAAACTA-GCGTCCT 15264

Haplotype A ..C................................C........-...C...

Haplotype C ..C................................C........-...C...

Haplotype D ..C................................C........-...C...

Pseudogene B TA..T....................A.....C..........T..-.T.....

Pseudogene E .......A..........T......A.T...C.G.G.C..G.T..AAT.....

Pseudogene F TG.....T.................A.....C....C..T..T..-AT.....

Pseudogene G .G................T.....TA.....C.....G....T..-AT.....

15271 15281 15291 15301 15311

| | | | |

NC_054160 15265 GATAGTATAAACATTACACTGGTCTTGTAAGCCAGAAATGAAATAACAGTT-T 15317

Haplotype A ..............................A....................-.

Haplotype C ..............................A....................-.

Haplotype D ..............................A....................-.

Pseudogene B ..............................A............C.......-.

Pseudogene E ..............................A............C..T.T..T.

Pseudogene F ..........G...................A...............T.T.---

Pseudogene G ..............................A...............T.T..-.

15320 15330 15340 15348 15358

| | | | |

NC_054160 15318 CTCAGGACATCAAGAAGGAAGGATCTA--CCCCCACCATCAACACCCAAAGCT 15367

Haplotype A .......................C...CC........................

Haplotype C .......................C...C-........................

Haplotype D .......................C...CC........................

Pseudogene B ....A..................C...CC........................

Pseudogene E .....A.T..T...............CCT............G...........

Pseudogene F ....................A.....CCT............G...........

Pseudogene G ..........................CCT............G...........

15368 15378 15388 15398 15408 15418

| | | | | |

NC_054160 15368 GATATTCTACTTAAACTACTTCTTGTACATAAATCTATATAGTACATAATACA 15420

Haplotype A ..C...............................T...........C......

Haplotype C ..C...............................T...........C......

Haplotype D ..C...............................T...........C......

Pseudogene B ..C...............................T...........C......

Pseudogene E ...G.....T........................T...C..............

Pseudogene F ..C...............................T...CC.............

Pseudogene G ..C...............................T...C..............

15428 15438 15448 15458 15468

| | | | |

NC_054160 15421 TTTATGTATATCGTACATTAAACTATATTCCCCTAGCATATAAGCAAGTACTA 15473

Haplotype A .A....................T..............................

Haplotype C .A....................T..............................

Haplotype D .A....................T..............................

Pseudogene B ......................T..............................

Pseudogene E ......................T..........A............T....--

Pseudogene F ............A.........T..........A............T......

Pseudogene G ......................T..............................

15478 15488 15498 15508 15518

| | | | |

NC_054160 15474 ACTATTAATTATATAAGACACTCATCTCTTCAATCAACATTACTCAACTACCA 15526

Haplotype A ....................T.--A.....T............CT..TC....

Haplotype C ....................T.--A.....T............CT..TC....

Haplotype D ....................T.--A.....T............CT..TC....

Pseudogene B ....................T..-AT..C.T..CT.....A..CT..T..A...

Pseudogene E --...C..............T.A.AT....T.-............CTTC.T-.

Pseudogene F ........C......G....T.ACA.....T.-............CTTC.T-.

Pseudogene G ...............G....T...AT......-.......C...TC-TCCT..

15528 15538 15548 15558 15568 15578

| | | | | |

NC_054160 15527 ACATGTCTATTATACCCAACTACTTAATTAATGTTATAAAGACATATCTGCGT 15579

Haplotype A G....C........TT...T..T..........CC....G.............

Haplotype C G....C........TT...T..T..........CC....G.............

Haplotype D .....C........TT...T..T..........CC....G.............

Pseudogene B .....G......C..T...T..T..........C..C.T...........T..

Pseudogene E C............GAT...T..T............C..CG...........A.

Pseudogene F .T..AC........GT...T..T............C..T.............C

Pseudogene G .....C.........T..GT..T............C..T..............

15588 15598 15608 15618 15628

| | | | |

NC_054160 15580 TATCTTACATACACCATTCTGTCATAAACTCTTCTCTTCCATACGACTATCCC 15632

Haplotype A .....................................................

Haplotype C .....................................................

Haplotype D .....................................................

Pseudogene B ....A................................................

Pseudogene E ............G.......................G....C....T...T..

Pseudogene F ...........................................T.........

Pseudogene G ............................TC..................T....

15638 15648 15658 15668 15678

| | | | |

NC_054160 15633 TGTCCCCATTTAATCCCTTGATCTATCATCCTCCGTGAAACCAACAACCCGCC 15685

Haplotype A CT....T.C................C...........................

Haplotype C CT....T.C................C...........................

Haplotype D CT....T..................C...........................

Pseudogene B CT......G...G............C...........................

Pseudogene E CT.T.T......G..TA........CA......T................A..

Pseudogene F CT...T.....GG..TA..A.....C.................G.........

Pseudogene G CT....T.C..GG..T...A.....C.................G.........

15688 15698 15708 15718 15727

| | | | |

NC_054160 15686 CACCCGTGCCCCTCTTCTCGCTCCGGGCCCATAC-AACTTGGGGGTGACTAAT 15737

Haplotype A .....A............................-..................

Haplotype C .....A............................-..................

Haplotype D .....A............................-..................

Pseudogene B .....A..........................G.-..................

Pseudogene E ....TA.......A.....A.........T..T.T..................

Pseudogene F .....AGC..........A..............TT..................

Pseudogene G ...T.............................TT...........A......

15747 15757 15767 15777 15786

| | | | |

NC_054160 15738 GTGAAACTTTACCAGGCATCTGGTTCTTACCTCAGGGCCATA-TATTGGTTCA 15789

Haplotype A ..........................................-..........

Haplotype C ..........................................-..........

Haplotype D ..........................................-..........

Pseudogene B ..........................................-..A.......

Pseudogene E ......T....G..................T...........T..A..T..T.

Pseudogene F ..............................T...........T..A.......

Pseudogene G ..........................................-..A.......

15796 15806 15816 15826

| | | |

NC_054160 15790 TCGTCCATACGTTCCCCTTAAATAAGACATCTCGATGGTACGGGT 15834

Haplotype A .............................................

Haplotype C .............................................

Haplotype D .............................................

Pseudogene B .............................................

Pseudogene E ..A.........................................C

Pseudogene F ..A..........................................

Pseudogene G .............................................

**Supplementary Table S1.** List of Arvicolinae mtDNA D-loop sequences used for the phylogenetic analysis

| Species name | **NCBI GenBank ID** | |  |
| --- | --- | --- | --- |
| *Alticola macrotis* | | MT381923.1:1500-16355 | |
| *Arvicola amphibius* | | MT381921.1:15000-16085 | |
| *Ellobius talpinus* | | NC_054610.1 and obtained in this work using the ArvF and ArvR primers | |
| *Ellobius tancrei* | | obtained in this work using the ArvF and ArvR primers | |
| *Eolagurus luteus* | | MT492448.1:15100-16354 | |
| *Lagurus lagurus* | | MT492449.1:15100-16362 | |
| *Myodes glareolus* | | NC_024538.1:15100-16353 | |

**Supplementary Table S2.** List of contigs from the *E. talpinus* genome database GCA_001685095.1 containing NUMTs.

|  | **Contig Name** | **Contig Length** | **NUMT length** | **NUMT start** | **NUMT end** | **mtDNA start** | **mtDNA end** | **mtDNA element** |
| --- | --- | --- | --- | --- | --- | --- | --- | --- |
| 1 | **LOJH01009070** | 28845 | 45 | 3 | 47 | 1 | 45 | *trnF* |
| 2 | **LOJH01078380** | 9381 | 46 | 3 | 49 | 1 | 45 | *trnF* |
| 3 | **LOJH01133877** | 4735 | 162 | 3975 | 3814 | 1 | 161 | *trnF, rrnS* |
| 4 | **LOJH01166511** | 3116 | 136 | 3011 | 2876 | 1 | 131 | *trnF, rrnS* |
| 5 | **LOJH01062936** | 10791 | 110 | 10680 | 10789 | 16 | 124 | *trnF, rrnS* |
| 6 | **LOJH01107285** | 6895 | 667 | 594 | 3 | 49 | 527 | *rrnS* |
| 7 | **LOJH01051319** | 23474 | 229 | 12304 | 12532 | 91 | 317 | *rrnS* |
| 8 | **LOJH01002123** | 45279 | 286 | 44176 | 44461 | 102 | 385 | *rrnS* |
| 9 | **LOJH01013929** | 24484 | 193 | 24291 | 24483 | 102 | 293 | *rrnS* |
| 10 | **LOJH01029675** | 17348 | 214 | 12997 | 13207 | 151 | 363 | *rrnS* |
| 11 | **LOJH01174855** | 3847 | 101 | 3746 | 3846 | 179 | 279 | *rrnS* |
| 12 | **LOJH01080144** | 8780 | 93 | 2805 | 2713 | 222 | 313 | *rrnS* |
| 13 | **LOJH01127242** | 6622 | 618 | 3 | 620 | 242 | 804 | *rrnS* |
| 14 | **LOJH01060256** | 19729 | 171 | 17991 | 18161 | 259 | 428 | *rrnS* |
| 15 | **LOJH01082636** | 8537 | 63 | 931 | 869 | 298 | 360 | *rrnS* |
| 16 | **LOJH01014372** | 24173 | 631 | 23562 | 24171 | 306 | 753 | *rrnS* |
| 17 | **LOJH01052348** | 12365 | 187 | 12178 | 12364 | 318 | 504 | *rrnS* |
| 18 | **LOJH01069707** | 9949 | 51 | 9947 | 9897 | 348 | 398 | *rrnS* |
| 19 | **LOJH01036094** | 15596 | 446 | 3 | 438 | 409 | 853 | *rrnS* |
| 20 | **LOJH01008703** | 36310 | 419 | 33500 | 33914 | 445 | 742 | *rrnS* |
| 21 | **LOJH01077222** | 15847 | 139 | 3 | 138 | 479 | 342 | *rrnS* |
| 22 | **LOJH01001272** | 67316 | 453 | 4006 | 3606 | 498 | 785 | *rrnS* |
| 23 | **LOJH01002789** | 41343 | 171 | 5849 | 6019 | 508 | 677 | *rrnS* |
| 24 | **LOJH01228719** | 1078 | 319 | 1076 | 767 | 546 | 863 | *rrnS* |
| 25 | **LOJH01162490** | 3292 | 160 | 3131 | 3290 | 586 | 745 | *rrnS* |
| 26 | **LOJH01001744** | 46671 | 79 | 3 | 81 | 602 | 680 | *rrnS* |
| 27 | **LOJH01206002** | 1668 | 107 | 3 | 109 | 605 | 710 | *rrnS* |
| 28 | **LOJH01001946** | 45498 | 197 | 3 | 199 | 683 | 879 | *rrnS* |
| 29 | **LOJH01001175** | 51218 | 214 | 22119 | 21906 | 696 | 909 | *rrnS* |
| 30 | **LOJH01119258** | 5622 | 167 | 5455 | 5621 | 731 | 897 | *rrnS* |
| 31 | **LOJH01210616** | 1537 | 315 | 316 | 2 | 732 | 1046 | *rrnS, trnV* |
| 32 | **LOJH01195174** | 2010 | 268 | 3 | 270 | 1022 | 1289 | *trnV, rrnL* |
| 33 | **LOJH01188849** | 2230 | 183 | 2046 | 2288 | 1151 | 1333 | *rrnL* |
| 34 | **LOJH01037236** | 15314 | 458 | 3381 | 3835 | 1216 | 1670 | *rrnL* |
| 35 | **LOJH01067429** | 10223 | 288 | 9939 | 10221 | 1220 | 1507 | *rrnL* |
| 36 | **LOJH01198849** | 2231 | 181 | 2046 | 2228 | 1331 | 1151 | *rrnL* |
| 37 | **LOJH01042702** | 18949 | 239 | 18 | 256 | 1352 | 1589 | *rrnL* |
| 38 | **LOJH01191208** | 2144 | 357 | 3 | 357 | 1354 | 1709 | *rrnL* |
| 39 | **LOJH01115659** | 6211 | 787 | 1908 | 1140 | 1385 | 2168 | *rrnL* |
| 40 | **LOJH01050595** | 12650 | 66 | 2866 | 2931 | 1395 | 1460 | *rrnL* |
| 41 | **LOJH01106773** | 6491 | 684 | 5499 | 4945 | 1436 | 2078 | *rrnL* |
| 42 | **LOJH01192053** | 2115 | 1587 | 1441 | 3 | 1458 | 1697 | *rrnL, trnL2* |
| 43 | **LOJH01080586** | 8738 | 371 | 3 | 370 | 1462 | 1830 | *rrnL* |
| 44 | **LOJH01091346** | 8050 | 2253 | 3 | 2255 | 1493 | 3798 | *rrnL, trnL2, ND1, trnI, trnQ* |
| 45 | **LOJH01017800** | 34297 | 1241 | 21566 | 22779 | 1507 | 2437 | *rrnL* |
| 46 | **LOJH01188478** | 2571 | 1571 | 1543 | 2 | 1531 | 2844 | *rrnL, trnL2, ND1* |
| 47 | **LOJH01030584** | 17066 | 1698 | 14439 | 12788 | 1561 | 6 | *trnF, rrnS, trnV, rrnL* |
| 48 | **LOJH01035804** | 18958 | 51 | 6991 | 7040 | 1581 | 1630 | *rrnL* |
| 49 | **LOJH01028236** | 17802 | 247 | 2 | 248 | 1750 | 1996 | *rrnL* |
| 50 | **LOJH01040409** | 14622 | 786 | 9118 | 9902 | 1758 | 2522 | *rrnL* |
| 51 | **LOJH01188605** | 3665 | 392 | 1192 | 1583 | 1766 | 2151 | *rrnL* |
| 52 | **LOJH01098553** | 7118 | 97 | 175 | 86 | 1856 | 1946 | *rrnL* |
| 53 | **LOJH01094208** | 7481 | 873 | 2094 | 1252 | 1890 | 2521 | *rrnL* |
| 54 | **LOJH01094878** | 7425 | 4536 | 1515 | 6050 | 2001 | 6613 | *rrnL, trnL2, ND1, trnI, trnQ, trnM, ND2, trnW, trnA, trnN, rep ori, trnC, trnY, COX1* |
| 55 | **LOJH01002076** | 66848 | 180 | 66668 | 66847 | 2003 | 2182 | *rrnL* |
| 56 | **LOJH01195086** | 2013 | 99 | 3 | 101 | 2006 | 2104 | *rrnL* |
| 57 | **LOJH01039449** | 20657 | 117 | 20539 | 20655 | 2120 | 2004 | *rrnL* |
| 58 | **LOJH01012010** | 35698 | 259 | 25920 | 26178 | 2419 | 2665 | *rrnL, trnL2* |
| 59 | **LOJH01057840** | 12396 | 303 | 12087 | 12389 | 2530 | 2832 | *rrnL, trnL2, ND1* |
| 60 | **LOJH01036772** | 15433 | 56 | 15376 | 15431 | 2564 | 2619 | *rrnL* |
| 61 | **LOJH01069523** | 15085 | 467 | 507 | 902 | 2567 | 2914 | *rrnL, trnL2, ND1* |
| 62 | **LOJH01115577** | 6208 | 1168 | 6207 | 5051 | 2770 | 3817 | *ND1, trnI, trnQ* |
| 63 | **LOJH01085369** | 8267 | 2286 | 6003 | 8266 | 3104 | 4960 | *ND1, trnI, trnQ, trnM, ND2, trnW* |
| 64 | **LOJH01009133** | 28782 | 1612 | 2391 | 4002 | 3209 | 1599 | *ND1, trnL2, rrnL* |
| 65 | **LOJH01000851** | 54978 | 223 | 22554 | 22776 | 3253 | 3475 | *ND1* |
| 66 | **LOJH01065541** | 10455 | 1085 | 2 | 1077 | 3553 | 4441 | *ND1, trnI, trnQ, trnM, ND2* |
| 67 | **LOJH01068681** | 10069 | 33 | 9250 | 9282 | 3785 | 3753 | *trnQ* |
| 68 | **LOJH01091346** | 8050 | 1652 | 2430 | 4081 | 3803 | 5443 | *trnQ, trnM, ND2, trnW, trnA, trnN, rep ori, trnC, trnY, COX1* |
| 69 | **LOJH01118836** | 5650 | 2392 | 3910 | 6314 | 3910 | 6313 | *COX1, trnS2, trnD, COX2, trnK* |
| 70 | **LOJH01007876** | 30359 | 3031 | 11120 | 13746 | 3964 | 6617 | *ND2, trnW, trnA, trnN,* rep ori*, trnC, trnY, COX1* |
| 71 | **LOJH01038931** | 14945 | 56 | 14894 | 14943 | 4303 | 4352 | *ND2* |
| 72 | **LOJH01118836** | 5650 | 2805 | 2844 | 5648 | 4386 | 1433 | *rrnL, trnL, ND1, trnI, trnQ, trnM, ND2* |
| 73 | **LOJH01115760** | 5858 | 224 | 5634 | 5857 | 4544 | 4767 | *ND2* |
| 74 | **LOJH01196672** | 1961 | 405 | 407 | 3 | 4549 | 4953 | *ND2, trnW* |
| 75 | **LOJH01157460** | 3521 | 483 | 2976 | 3454 | 4561 | 5043 | *ND2, trnW, trnA* |
| 76 | **LOJH01125826** | 5209 | 290 | 3 | 292 | 4621 | 4910 | *ND2* |
| 77 | **LOJH01142877** | 4245 | 707 | 2443 | 3144 | 4626 | 5327 | *ND2, trnW, trnA, trnN, rep ori, trnC, trnY, COX1* |
| 78 | **LOJH01131954** | 9217 | 575 | 434 | 1008 | 4736 | 5310 | *ND2, trnW, trnA, trnN, rep ori, trnC, trnY, COX1* |
| 79 | **LOJH01205309** | 1688 | 132 | 134 | 3 | 5005 | 5136 | *trnA, trnN, rep ori* |
| 80 | **LOJH01191906** | 2120 | 721 | 3 | 711 | 5068 | 5781 | *trnN,* rep ori*, trnC, trnY, COX1* |
| 81 | **LOJH01052963** | 12262 | 413 | 11386 | 11776 | 5079 | 5417 | *rep ori, trnN, trnC, trnY, COX1* |
| 82 | **LOJH01173053** | 2844 | 243 | 9 | 251 | 5112 | 5354 | *trnN, rep ori, trnC, trnY, COX1* |
| 83 | **LOJH01112352** | 6087 | 167 | 4794 | 4958 | 5150 | 5315 | *rep ori, trnC, trnY, COX1* |
| 84 | **LOJH01057721** | 11514 | 228 | 9540 | 9766 | 5172 | 4946 | *trnW, trnA, trnN, rep ori, trnC* |
| 85 | **LOJH01032790** | 16453 | 1015 | 8 | 1019 | 5233 | 6161 | *trnY, COX1* |
| 86 | **LOJH01229498** | 1062 | 367 | 3 | 369 | 5260 | 5626 | *trnY, COX1* |
| 87 | **LOJH01106085** | 6539 | 150 | 6537 | 6880 | 5301 | 5450 | *COX1* |
| 88 | **LOJH01071429** | 9739 | 241 | 9737 | 9497 | 5303 | 5541 | *COX1* |
| 89 | **LOJH01036148** | 15585 | 999 | 4 | 1002 | 5308 | 6303 | *COX1* |
| 90 | **LOJH01136509** | 4594 | 213 | 4380 | 4592 | 5450 | 5238 | *trnY, COX1* |
| 91 | **LOJH01031780** | 26568 | 1081 | 11346 | 12407 | 5454 | 4378 | *ND2, trnW, rep ori, trnA, trnN, trnY, COX1* |
| 92 | **LOJH01038399** | 15059 | 1576 | 13490 | 15057 | 5476 | 6871 | *COX1, trnS2* |
| 93 | **LOJH01079493** | 8848 | 74 | 6833 | 6760 | 5615 | 5688 | *COX1* |
| 94 | **LOJH01009133** | 28782 | 620 | 2352 | 1738 | 5754 | 6373 | *COX1* |
| 95 | **LOJH01069100** | 15990 | 1429 | 1554 | 2963 | 5759 | 4401 | *ND2, trnW, trnA, trnN*, rep ori*, trnC, trnY, COX1* |
| 96 | **LOJH01087894** | 11456 | 1541 | 6981 | 8443 | 5791 | 6757 | *COX1* |
| 97 | **LOJH01000262** | 70077 | 83 | 27102 | 27184 | 6379 | 6461 | *COX1* |
| 98 | **LOJH01010421** | 27451 | 256 | 3 | 258 | 6600 | 6854 | *trnS2, COX1* |
| 99 | **LOJH01152741** | 3748 | 316 | 3205 | 3520 | 6614 | 7840 | *trnK, ATP8* |
| 100 | **LOJH01211564** | 1510 | 565 | 1507 | 947 | 6631 | 7047 | *COX1, trnS, trnD, COX2* |
| 101 | **LOJH01009498** | 50855 | 302 | 9933 | 10194 | 6646 | 6879 | *COX1, trnS2* |
| 102 | **LOJH01008122** | 46600 | 314 | 36159 | 36472 | 6656 | 6969 | *COX1, trnS2, trnD* |
| 103 | **LOJH01028962** | 24086 | 685 | 18853 | 19533 | 6724 | 7259 | *COX1, trnS2,trnD, COX2* |
| 104 | **LOJH01009964** | 29948 | 668 | 26555 | 27213 | 6725 | 6148 | *COX1* |
| 105 | **LOJH01162682** | 3556 | 2943 | 23 | 2311 | 6775 | 9542 | *COX1, trnS2, trnD, COX2, trnK, ATP8, ATP6, COX3, trnG, ND3* |
| 106 | **LOJH01230986** | 1032 | 209 | 652 | 444 | 6792 | 6997 | *COX1, trnS2, trnD, COX2* |
| 107 | **LOJH01165017** | 3182 | 409 | 2772 | 3180 | 6829 | 7237 | *COX1, trnS2, trnD, COX2* |
| 108 | **LOJH01009791** | 28084 | 295 | 23826 | 24107 | 6951 | 7226 | *trnD, COX2* |
| 109 | **LOJH01047207** | 13257 | 371 | 12885 | 13255 | 7133 | 7503 | *COX2* |
| 110 | **LOJH01159440** | 3428 | 44 | 3420 | 3377 | 7189 | 7232 | *COX2* |
| 111 | **LOJH01129715** | 4978 | 429 | 3 | 431 | 7673 | 8101 | *trnK, ATP8, ATP6* |
| 112 | **LOJH01059647** | 11235 | 3121 | 8120 | 11172 | 7685 | 10794 | *trnK, ATP8, ATP6, COX3, trnG, ND3, trnR, ND4L, ND4* |
| 113 | **LOJH01083867** | 10331 | 462 | 3 | 464 | 7801 | 8262 | *ATP8, ATP6* |
| 114 | **LOJH01112010** | 6110 | 695 | 6108 | 5418 | 7895 | 8515 | *ATP6* |
| 115 | **LOJH01028777** | 33628 | 160 | 7172 | 7331 | 8275 | 8116 | *ATP6* |
| 116 | **LOJH01142279** | 4278 | 483 | 4271 | 3789 | 8440 | 8922 | *ATP6, COX3* |
| 117 | **LOJH01155116** | 3633 | 535 | 917 | 392 | 8513 | 8882 | *ATP6, COX3* |
| 118 | **LOJH01217014** | 1653 | 850 | 11 | 856 | 8961 | 9729 | *COX3, trnG, ND3* |
| 119 | **LOJH01023134** | 21632 | 1769 | 19776 | 21512 | 9312 | 11009 | *ND4, ND4L, trnR, ND3, trnG, COX3* |
| 120 | **LOJH01033650** | 16229 | 94 | 3 | 96 | 9384 | 9477 | *trnG, ND3* |
| 121 | **LOJH01016980** | 26285 | 1437 | 2959 | 4317 | 9886 | 8509 | *ATP6, COX3, trnG, ND3, trnR, ND4L* |
| 122 | **LOJH01047587** | 13919 | 373 | 5 | 375 | 10177 | 10544 | *ND4* |
| 123 | **LOJH01110989** | 6182 | 737 | 6 | 742 | 10313 | 11049 | *ND4* |
| 124 | **LOJH01197810** | 3351 | 3560 | 3340 | 3 | 10495 | 14004 | *ND4, trnH, trnS1, trnL1, ND5, ND6, trnE, CYTB* |
| 125 | **LOJH01076753** | 10021 | 1249 | 8 | 1243 | 10529 | 9309 | *trnG, ND3, trnR, ND4L, ND4, trnH* |
| 126 | **LOJH01070646** | 9834 | 180 | 5826 | 5647 | 10660 | 10843 | *ND4* |
| 127 | **LOJH01152185** | 5863 | 4193 | 1719 | 5858 | 10795 | 14414 | *ND4, trnH, trnS1, trnL1, ND5, ND6, trnE, CYTB* |
| 128 | **LOJH01127637** | 5100 | 2210 | 2973 | 5098 | 11497 | 13458 | *ND4, trnH, trnS1, trnL1, ND5,* |
| 129 | **LOJH01013982** | 24451 | 1396 | 20992 | 22379 | 11548 | 12939 | *trnH, trnS1, trnL1, ND5* |
| 130 | **LOJH01116896** | 10000 | 3257 | 6788 | 9997 | 11563 | 14310 | *trnH, trnS1, trnL1, ND5, ND6, trnE, CYTB* |
| 131 | **LOJH01000523** | 60739 | 83 | 23151 | 23069 | 11565 | 11647 | *trnH, trnS1, trnL* |
| 132 | **LOJH01079530** | 8844 | 3971 | 4911 | 8842 | 11585 | 15140 | *trnS1, trnL1, ND5, ND6, trnE, CYTB* |
| 133 | **LOJH01002023** | 49718 | 156 | 2 | 157 | 11587 | 11432 | *ND4, trnH, trnS1* |
| 134 | **LOJH01029677** | 17348 | 91 | 11365 | 11453 | 11592 | 11502 | *ND4, trnH, trnS1* |
| 135 | **LOJH01126046** | 7548 | 328 | 5145 | 5303 | 11661 | 11960 | *trnL1, ND5* |
| 136 | **LOJH01112811** | 7320 | 1594 | 1594 | 3 | 11734 | 13326 | *ND5* |
| 137 | **LOJH01011730** | 26213 | 401 | 17733 | 18131 | 11827 | 12219 | *ND5* |
| 138 | **LOJH01028551** | 17707 | 855 | 16852 | 17705 | 12604 | 13458 | *ND5* |
| 139 | **LOJH01147659** | 4000 | 145 | 3782 | 3926 | 12731 | 12875 | *ND5* |
| 140 | **LOJH01140977** | 4348 | 311 | 313 | 3 | 12871 | 13181 | *ND5* |
| 141 | **LOJH01087719** | 8050 | 323 | 7726 | 8048 | 12977 | 13299 | *ND5* |
| 142 | **LOJH01083907** | 8412 | 264 | 267 | 4 | 13022 | 13285 | *ND5* |
| 143 | **LOJH01007150** | 45426 | 505 | 28675 | 29175 | 13117 | 13621 | *ND5, ND6* |
| 144 | **LOJH01065472** | 10462 | 312 | 10149 | 10460 | 13250 | 13561 | *ND6, ND5* |
| 145 | **LOJH01025898** | 18584 | 435 | 2069 | 2489 | 13255 | 13645 | *ND6, ND5* |
| 146 | **LOJH01218015** | 1334 | 684 | 622 | 9 | 13431 | 13942 | *ND5, ND6* |
| 147 | **LOJH01027858** | 17934 | 1779 | 3021 | 4683 | 13631 | 15401 | *ND6, trnE, CYTB, trnT, trnP* |
| 148 | **LOJH01222549** | 1604 | 593 | 889 | 1407 | 13879 | 14413 | *ND6, trnE, CYTB* |
| 149 | **LOJH01064739** | 10945 | 167 | 7568 | 7720 | 14000 | 14166 | *ND6, trnE, CYTB* |
| 150 | **LOJH01184598** | 2386 | 192 | 194 | 3 | 14159 | 14350 | *CYTB* |
| 151 | **LOJH01204008** | 1728 | 202 | 204 | 3 | 14206 | 14407 | *CYTB* |
| 152 | **LOJH01000724** | 76214 | 394 | 48711 | 49101 | 14279 | 14499 | *CytB* |
| 153 | **LOJH01006316** | 32638 | 221 | 7 | 226 | 14313 | 14533 | *CYTB* |
| 154 | **LOJH01111099** | 6174 | 802 | 6172 | 5371 | 14414 | 15215 | *CYTB* |
| 155 | **LOJH01155328** | 3623 | 139 | 1731 | 1593 | 14523 | 14661 | *CYTB* |
| 156 | **LOJH01186584** | 3965 | 721 | 1264 | 1980 | 14613 | 15262 | *CYTB* |
| 157 | **LOJH01099250** | 7059 | 252 | 11 | 262 | 14725 | 14976 | *CYTB* |
| 158 | **LOJH01055173** | 21576 | 256 | 3 | 258 | 15029 | 15284 | *trnT, CYTB* |
| 159 | **LOJH01065826** | 10418 | 802 | 1247 | 14273 | 15070 | 14882 | *CYTB* |
| 160 | **LOJH01111898** | 12186 | 273 | 3 | 274 | 15085 | 15352 | *CYTB, trnT, trnP* |
| 161 | **LOJH01013788** | 24587 | 245 | 19831 | 20075 | 15103 | 15347 | *CYTB, trnT, trnP* |
| 162 | **LOJH01121637** | 5467 | 204 | 3 | 206 | 15143 | 15346 | *CYTB, trnT, trnP* |
| 163 | **LOJH01150395** | 3862 | 155 | 3 | 157 | 15265 | 15418 | *trnT, trnP*, D-loop |
| 164 | **LOJH01246111** | 730 | 68 | 407 | 474 | 15270 | 15337 | *trnT, trnP* |
| 165 | **LOJH01128614** | 9691 | 172 | 9519 | 9690 | 15337 | 15167 | *trnP, trnT, CYTB* |
| 166 | **LOJH01152243** | 3772 | 69 | 3702 | 3770 | 15339 | 15271 | *trnP, trnT* |
| 167 | **LOJH01148643** | 3950 | 221 | 3727 | 3947 | 15348 | 15129 | *trnP,trnT, CYTB* |
| 168 | **LOJH01204536** | 1712 | 355 | 1363 | 1711 | 15351 | 15705 | *trnP*, D-loop |
| 169 | **LOJH01175701** | 3967 | 130 | 3 | 131 | 15360 | 15485 | D-loop |
| 170 | **LOJH01108992** | 8953 | 380 | 8576 | 8951 | 15366 | 15110 | *trnP, trnT, CYTB* |
| 171 | **LOJH01095061** | 16401 | 233 | 93 | 279 | 15375 | 15598 | *trnP,* D-loop |
| 172 | **LOJH01005386** | 34263 | 101 | 14730 | 14631 | 15391 | 15491 | D-loop |
| 173 | **LOJH01048570** | 13007 | 247 | 12757 | 13004 | 15397 | 15643 | D-loop |
| 174 | **LOJH01029690** | 24307 | 204 | 5167 | 5368 | 15411 | 15614 | D-loop |
| 175 | **LOJH01049918** | 12772 | 634 | 4511 | 3884 | 15411 | 15849 | D-loop |
| 176 | **LOJH01038625** | 15011 | 700 | 15009 | 14347 | 15419 | 16106 | D-loop |
| 177 | **LOJH01079492** | 8848 | 408 | 3 | 408 | 15459 | 15865 | D-loop |
| 178 | **LOJH01021980** | 29064 | 581 | 5170 | 5609 | 15508 | 16079 | D-loop |
| 179 | **LOJH01210837** | 1530 | 109 | 3 | 105 | 15559 | 15451 | *D-loop* |
| 180 | **LOJH01138175** | 4500 | 862 | 3 | 1164 | 15631 | 14289 | D-loop*, trnP, trnT, CYTB* |
| 181 | **LOJH01019880** | 21013 | 477 | 20544 | 20977 | 15684 | 16139 | *D-loop* |
| 182 | **LOJH01100772** | 6937 | 201 | 6736 | 6936 | 15722 | 15915 | *D-loop* |
| 183 | **LOJH01143691** | 4202 | 331 | 2 | 330 | 15726 | 15397 | *D-loop* |
| 184 | **LOJH01152972** | 6671 | 554 | 3 | 552 | 15771 | 15221 | D-loop*, trnP, trnT, CYTB* |
| 185 | **LOJH01114898** | 6288 | 523 | 5768 | 6286 | 15774 | 15442 | D-loop |
| 186 | **LOJH01024859** | 18962 | 248 | 251 | 5 | 15783 | 16025 | D-loop |
| 187 | **LOJH01087830** | 8040 | 3098 | 3 | 2978 | 15800 | 13055 | D-loop*, trnP, trnT, CYTB, trnE, ND6, ND5* |
| 188 | **LOJH01123952** | 5322 | 241 | 3 | 243 | 15830 | 16070 | D-loop |
| 189 | **LOJH01078322** | 9284 | 134 | 9149 | 9282 | 15873 | 15741 | D-loop |
| 190 | **LOJH01055366** | 11879 | 1706 | 9077 | 10770 | 15979 | 14280 | D-loop*, trnP, trnT, CYTB* |
| 191 | **LOJH01078005** | 9004 | 112 | 3 | 114 | 16033 | 16144 | D-loop |
| 192 | **LOJH01163949** | 3229 | 273 | 1517 | 1787 | 16103 | 16364 | D-loop |
| 193 | **LOJH01008097** | 30048 | 892 | 59 | 896 | 16156 | 15741 | D-loop |
| 194 | **LOJH01000957** | 53681 | 154 | 50414 | 50261 | 16170 | 16232 | D-loop |
| 195 | **LOJH01107520** | 6436 | 31 | 3472 | 3442 | 16182 | 16212 | D-loop |

**Supplementary Figure S4.** Size distribution of NUMTs in *E. talpinus*.

**Supplementary Figure S5.** Three examples of split reads comprising both a fragment of mitochondrial DNA (highlighted in yellow) and a fragment of nuclear genomic DNA (highlighted in blue)

SRR3497471.144330697 ATAAGACATCTCGATGGTACGGGTCTAATCAGCCCATGCCAACATGTTCTACTTTTATTAAAAAGATTTGCACAATTTACTAATTTTATAACCAACCCACA

SRR3497471.278589963

TCAGGGCCATATAATGGTTCATCGTCCATACGTTCCCCTTAAATAAGACATCTCGATGGTACGGGTCTAATCAGCCCATGCCAACATGTTCTACTTTTATT

SRR3497471.187978918

ATCGTCCATACGTTCCCCTTAAATAAGACATCTCGATGGTACGGGTCTAATCAGCCCATGCCAACATGTTCTACTTTTATTAAAAAGATTTGCACAATTTA
